# Motivation and Habits of a Wild Boar-Hunting Community

**DOI:** 10.3390/ani14131940

**Published:** 2024-06-30

**Authors:** Vasileios J. Kontsiotis, Apostolos Polychronidis, Vasilios Liordos

**Affiliations:** Department of Forest and Natural Environment Sciences, Democritus University of Thrace, P.O. Box 172, 66100 Drama, Greece; vkontsiotis@neclir.duth.gr (V.J.K.); lotis96@yahoo.gr (A.P.)

**Keywords:** suidae, questionnaire survey, hunting practice, consumptive activity, leisure, outdoors

## Abstract

**Simple Summary:**

The wild boar, found on all the continents except Antarctica, is among the most valuable game species worldwide. However, the recent increase in their numbers has caused negative impacts, such as biodiversity loss, crop destruction, vehicle collisions, and disease transmission. Therefore, wildlife managers need to apply appropriate strategies to achieve three goals: providing sufficient game, mitigating impacts, and securing viable populations. Such efforts cannot be successful without the consent and participation of key stakeholders such as wild boar hunters. We asked 134 hunters of the Evros Prefecture in Greece, a wild boar stronghold, about their practice of hunting and their motivation for hunting. Hunting is carried out in groups, while the number of days out, kilometers traveled, and weekly expenses for hunting in a hunting season suggest the strong attachment of hunters to their game. Hunting is mostly considered a recreational activity (offering contact with nature and excitement, enjoyment of wildlife, bonding with fellow hunters and their dogs, and an opportunity for improving physical shape and reducing stress), although utility is also an important motivation (feeling useful for the family, helping wild boar control and the local economy). Hunters with strong recreation and utility motivations want wild boar populations to further increase. This information can be incorporated into education and outreach programs, to educate hunters about the risks with which increased wild boar numbers are associated. Educated hunters will then offer valuable contributions to effectively manage the situation.

**Abstract:**

The wild boar (*Sus scrofa*) is a widespread ungulate, the populations of which have recently increased throughout most of its range. This increase has caused negative impacts on ecosystems, biodiversity, and society. Nowadays, the wild boar is considered both a valuable game and a pest. Wildlife managers need to know the habits and motivations of wild boar hunters, a key stakeholder group, for effectively managing this controversial mammal. We carried out face-to-face interviews with 134 wild boar hunters in the Evros Prefecture, in the Region of Eastern Macedonia and Thrace, northern Greece to determine their hunting habits and their motivation for hunting. Most hunters owned a hunting dog (84.3%), hunted in groups of eight to nine people for 48 days, traveled 60 km, stayed outdoors for 4 nights, and spent weekly EUR 61 on average in each hunting season. Two motivations for wild boar hunting were prominent among the hunters (assessed on a 5-point scale; 1 = strongly disagree to 5 = strongly agree): a strong recreation motivation (hunting as a recreational activity; mean score 4.159 ± 1.144 SD) and a considerable utility motivation (hunting for its use values; 3.404 ± 1.11). Both recreation and utility motivations were positively associated with the preference for further increases in wild boar populations. Sociodemographic characteristics and hunting habits variously affected motivations and preferred future population trends. The findings revealed specific habits and strong motivations among hunters. Such findings will be useful for designing and implementing education and outreach programs for informing hunters about the negative impacts of wild boars and the need for their control. The participation of hunters in the management process will be critical for its success.

## 1. Introduction

Hunting has been an integral part of human societies since the dawn of civilization [1]. It has provided subsistence, such as food, clothing, and tools, for ages. In modern times, hunting has changed from a life-supporting to a recreational activity, especially in Western societies [2,3]. In general, people’s well-being has increased in the last 200 years, and societies have become urbanized. This has led to the rise of nature and animal ethics, and the adoption of more moralistic values toward wildlife, seeing them as part of one’s extended family, deserving of rights like humans [4]. These behavioral changes have been responsible for the decline in hunting participation in Western societies [5,6,7,8]. However, despite this decline, hunting is still a widespread and important socioeconomic activity [9,10]. Within this framework, the good practice of hunting can offer several benefits to people and nature. It has been used as a management tool for controlling harmful invasive alien species and overabundant native populations for which natural predators have disappeared [11,12,13]. Hunters help boost local and national economies through the payment of hunting fees and the use of equipment, clothing, transport, and accommodation services for pursuing their favorite activity [14,15]. Funds from hunting fees have been used for the conservation of vulnerable wildlife populations [16,17]. Such interventions promote ecosystem and human health and well-being.

Wildlife management is rarely successful without integrating human dimensions [18]. Managers need to know wildlife populations but also the preferences and motivations of key stakeholders of wildlife management, such as hunters, to be able to efficiently apply conservation and management strategies for the benefit of both people and wildlife [19].

The wild boar (*Sus scrofa*) is one of the most widespread mammals in the world, with its natural distribution ranging from western Europe to eastern Russia, Japan, and Southeast Asia, having been introduced into the Americas, Africa, and Oceania [20,21]. Despite being an ungulate, the wild boar has a high reproductive output, from their first year, of 5 piglets per litter on average and up to 13 annually, like that of small passerines and rodents [22]. It is also highly adaptable to changing environmental conditions, and its populations can greatly increase under favorable conditions [23]. Populations of wild boar have recently highly increased and expanded across Europe [24,25]. Besides natural and agricultural areas, wild boars have also expanded in urban parks, attracted by the increased food and water availability [26]. These trends have been attributed to factors such as milder winters due to climate change [27], an increase in agricultural land [28] and artificial feeding [29], and a decrease in natural predators [24,30]. The ever-increasing wild boar population has exacerbated or introduced negative impacts, such as damage to crops such as corn, potatoes, and fruits [31,32], hybridization with domestic pigs [33], issues with forest ecosystems [34] and biodiversity [35], collisions with vehicles [36,37], and the transmission of diseases such as African swine fever, bovine tuberculosis, trichinosis, salmonella, and leptospirosis [38,39,40]. The increased risk of disease transmission and the consequent need for precautionary actions such as biosafety measures, health monitoring, and movement restrictions incur additional economic costs to cattle farms [41].

The wild boar is among the most popular game species worldwide [42]. Wild boar hunting can be a single hunt, where a hunter waits from a post, often using bait [43], or a driven hunt, where hunters are evenly distributed at several posts at the periphery of an area and hog hounds flush wild boars from their cover toward the hunters in the periphery [44,45]. Due to its popularity, wild boar hunting has important implications regarding the hunters’ well-being and the socioeconomic activities associated with hunting [9,17]. Wild boar hunters usually reject the management of wild boar impacts using lethal strategies, highly preferring the use of non-lethal strategies [46,47]. The motivation for hunting has been explored for hunters in general, but not for wild boar hunters as a distinct group [12,15,17]. Wild boar hunters usually hunt in groups, and their success and safety depend on a high degree of cooperation [43,44]. The specific requirements of wild boar hunting indicate the importance of studying the motivations of wild boar hunters separately.

Its large population increase has turned the wild boar into a controversial animal. On the one hand, it is considered a “friend”, a valuable game offering psychological and physiological benefits to hunters and economic benefits to society [9,12,15,17]. On the other hand, it is considered an “enemy”, a pest negatively affecting forested, agricultural, and urban lands, and posing a threat to biodiversity and people [31,32,33,34,35,36,37,38,39,40]. Wildlife managers need to implement strategies that will both minimize its negative impacts and secure healthy wild boar populations for allowing hunting. This is not an easy task, requiring the collaboration of key stakeholders such as wild boar hunters [46,47]. Knowledge of the practice of hunting and the motivations of wild boar hunters will allow wildlife managers to integrate this key group into the management of this controversial animal.

Wild boar is also among the favorite game in Greece, especially in its population stronghold, northern Greece [48]. The attitudes of hunters in general have been studied in Greece [12,14,17], but not the attitudes of wild boar hunters in particular. Tsachalidis and Hadjisterkotis [48] studied the socioeconomic trends and aspects of wild boar-hunting practice, but not hunters’ attitudes. This study aimed to reveal the motivations and habits of wild boar hunters in a wild boar hotspot in northern Greece, the Evros Prefecture.

## 2. Materials and Methods

### 2.1. Study Area

The study was carried out in the Evros Prefecture, Region of Eastern Macedonia and Thrace, northern Greece (40°59′55″ Ν, 26°00′30″ E). The Evros River forms the natural border of Greece with Turkey to the East. The prefecture is largely covered by habitats suitable for wild boars. The southern part is covered by large wetlands formed by the Evros Delta. Oak forests (dominated by Hungarian oak *Quercus frainetto*), mixed oak–pine forests (Hungarian oak, Calabrian pine *Pinus brutia*, European black pine *Pinus nigra*), pine forests (Calabrian pine, European black pine), and maquis (kermes oak *Quercus coccifera*, green olive tree *Phillyrea latifolia*) dominate the rest of the Prefecture. About 3000 hunting licenses are issued annually in the Prefecture, with an estimated 1000 wild boar hunters among them. The population of wild boar has increased in the last two decades, and wild boar kills have evolved from about 700 to 800–1200 in a hunting season. Wild boar hunting is allowed from 15 September to 20 January, on Wednesdays, Saturdays, and Sundays without bag restrictions, totaling 54 days in each hunting season.

### 2.2. Sample Collection

Questionnaires were collected through face-to-face surveys with wild boar hunters, between August and October 2020, a period when COVID-19 restrictions had relaxed. A pretest of the survey (*n* = 30 random people) was conducted to test question clarity and completion time. Hunters were approached in hunting clubs and in the Forest Service offices they visited to be issued hunting licenses. The researcher (A.P.) asked hunters about the game they hunted and then asked wild boar hunters to participate in the survey by reading and responding to questions in the questionnaire, with the help of the researcher if required (respondent-completed survey; [49]). It took respondents 30 min on average to complete the questionnaire.

### 2.3. Questionnaire Development

The questionnaire consisted of two parts. The survey participants were first asked about their habits and preferences related to wild boar hunting and their sociodemographic characteristics. Questions included hunting experience, their hunting group’s size, hunting dog ownership, days spent hunting, nights they stayed outdoors, weekly hunting expenses, maximum travel distance, commitment to hunting, and current and preferred future wild boar population trends. Sociodemographic characteristics included age, level of education, and income. See Table 1 for definitions and descriptive statistics.

In the second part of the questionnaire, the survey participants were asked about their motivations for hunting, using 12 motive statements, such as “I go wild boar hunting because it brings me to close contact with nature”. Participants were asked to assess how much they agreed or disagreed with each motive statement on a 5-point scale: “strongly disagree” (1), “disagree” (2), “neither” (3), “agree” (4), or “strongly agree” (5).

### 2.4. Data Analysis

An exploratory factor analysis (principal components with varimax rotation) was performed to determine meaningful motivation dimensions based on the 12 motive statements. The eigenvalue criterion of ≥1 was used for factor inclusion. Factor reliability was assessed with Cronbach’s alpha, with α > 0.7 determining that the statements included in a factor consistently measured the construct [50].

The relationships between the determined motivation dimensions and preferred future wild boar trends and hunters’ habits and sociodemographic characteristics were assessed with multiple linear regression models. A variance inflation factor (VIF <5) and Spearman correlation (*r_s_* < 0.7) were used to assess multicollinearity. The VIFs were <2.5, except for age (5.4) and hunting experience (4.1). The correlations were <0.557, except for the correlation between age and hunting experience (*r*_s_ = 0.767). Based on multicollinearity criteria, we removed age from the regression analyses. All the VIFs were <1.8 and *r*_s_ < 0.557 after removing age.

All statistical analyses were performed with SPSS Statistics (version 21.0, IBM Corp., New York, NY, USA, 2012). The significance level was set at *α* = 0.05.

## 3. Results

### 3.1. Description of Wild Boar-Hunting Community

We collected fully completed questionnaires from 137 wild boar hunters (93% response rate). Of these, 134 were men and 3 were women, the latter being removed from further analyses, resulting in an effective sample of *n* = 134 hunters. The mean hunter was about 42 years old, with low education, EUR 19,700 annual household income, and 15 years of hunting experience (Table 1).

Wild boar hunters in Evros pursue their pastime in groups of eight to nine people on average, and most of them own a specialized hunting dog (84.3%). On average, they hunt their favorite game for about 48 days, travel 60 km, and stay outdoors for 4 nights during the hunting season. They also spend a weekly average of EUR 61 on their pastime. Overall, the wild boar hunters were highly committed to their pastime. Most hunters (81.4%) stated that they would miss it if they had to stop hunting, with 67.9% of them finding it irreplaceable.

Most hunters believed that wild boar numbers have increased (59.0%), while about 20% thought they are stable or have decreased. On the other hand, hunters were divided about their preferences concerning future wild boar population trends. Some stated that wild boar populations should increase (38.1%), others that they should decrease (32.1%), and others that they should remain stable (29.9%).

### 3.2. Motivation for Wild Boar Hunting

**Figure 1:**
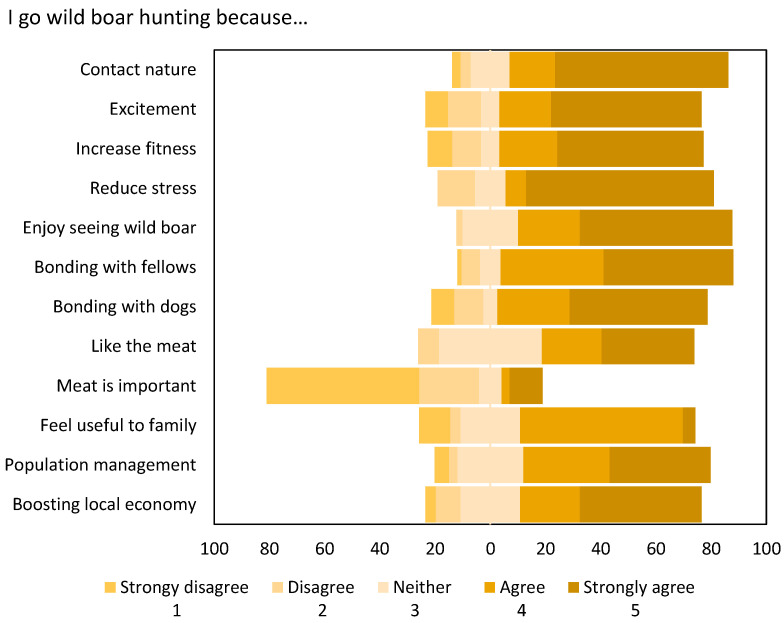
Wild boar hunters’ (*n* = 134) percentage responses to 12 motivations for hunting statements. See Table 2 for the full wording of the statements.

Most of the hunters (55.2–84.3%) stated that they go wild boar hunting because it allows for contact with nature, enjoying wildlife, and bonding with fellow hunters and their dogs, offers excitement, improves their physical shape, helps reduce daily stress, they like game meat, it makes them feel useful, and it helps population management and the local economy (Figure 1). On the other hand, most hunters did not consider wild boar meat an important subsistence for their family (76.9%).

Two motivations for wild boar-hunting dimensions with eigenvalues larger than 1 were determined by the exploratory factor analysis (eigenvalues 3.9 and 2.4; Table 2). The first, the “recreation dimension” indicated a strong recreation motivation among the wild boar hunters (mean score 4.159 ± 1.144 SD). The second, the “utility dimension” (3.404 ± 1.11) indicated that wild boar hunting has a considerable use value for hunters. The recreation and utility dimensions explained 43.3% and 26.1% of the variance, respectively. Also, the construct reliability was high for both dimensions (α > 0.7).

### 3.3. Effects of Habits and Sociodemographics on Motivation

The more educated hunters (*p* = 0.006), with higher income (*p* = 0.027), more committed to hunting (*p* < 0.001), who hunted in larger groups (*p* = 0.001), for more days (*p* < 0.001), traveled more (*p* = 0.009) and spent more (*p* < 0.001) on hunting wild boars were more highly motivated to hunt for recreation than the less educated hunters, with lower income, less committed to hunting, who hunted in smaller groups, for fewer days, traveled less, and spent less on hunting wild boars (Table 3).

The less educated hunters (*p* = 0.020), with lower income (*p* = 0.013), who believed that wild boar populations have increased (*p* = 0.001), stayed fewer nights outdoors (*p* = 0.001), and traveled less (*p* = 0.022) for hunting wild boars were more highly motivated to hunt for use values than the more educated hunters, with higher income, who believed that wild boar populations have decreased, stayed more nights outdoors, and traveled more for hunting wild boars (Table 3).

Both recreation (*p* < 0.001) and utility (*p* < 0.001) motivations were positively associated with a preference for future wild boar population increase (Table 3). Educational level (*p* < 0.001), hunting experience (*p* < 0.001), and current population trends (*p* < 0.001) were negatively, and committed to hunting (*p* = 0.040) positively, associated with a preference for future wild boar population increase.

## 4. Discussion

### 4.1. Profile and Habits of Wild Boar Hunters

Wild boar hunters are generally in early middle age, with a low educational level and considerable hunting experience. Tsachalidis and Hadjisterkotis [48] compared the sociodemographic characteristics of wild boar hunters between 1993 and 2002 in the Drama and Kavala Prefectures, northern Greece. They reported, similar to our findings, a mean age of 37 to 42 years, low education levels (85–100%), and between 12 and 17 years of hunting experience for wild boar hunters. Most wild boar hunters in Portugal were between 30 and 50 years old [51]. In Europe, hunters are mostly over 50 years old, of primary or secondary education, and with more than 10 years of hunting experience [52,53,54]. The difference in age between wild boar hunters and other hunters might be explained by the nature of wild boar hunting, which demands both responsibility, which comes with age, and stamina, characteristic of younger people. However, more research is necessary to resolve this issue.

We interviewed three women who were excluded from the analysis due to the small sample size. The exact number of women hunters in Greece is not known, but it is considered small, as previous studies have failed to include women [12,14,17,47,48]. Women hunters were not found in Antalya, Turkey, also [52]. However, in other European countries, the proportion of women hunters varied from <1% (Italy, Latvia, Croatia) to 10.5% (UK) [52,53,54,55].

Most of the wild boar hunters of Drama and Kavala declared an annual personal income of less than EUR 10,000 (94.6% in 1993 and 84.6% in 2002 in Drama; 83.3% in 1993 and 95.0% in 2002 in Kavala) [48]. These findings cannot be compared with ours because we asked for the annual household income. The wild boar hunters of Drama and Kavala hunted for about 25 to 45 days and traveled from 54 to 78 km on average during the hunting season. These findings are similar to ours, suggesting that the expenses of the hunters of Drama and Kaval for hunting wild boar were similar to those of Evros.

The wild boar hunters stated that they are highly committed to their favorite pastime. This statement can also be inferred by the high number of days that they went out hunting (88.9% of those when wild boar hunting was permitted). Hunters from central Greece and north Sweden also stated a strong commitment to hunting (74.5% of hunters in both samples) [14,56]. In general, hunters are among the outdoor recreation groups more attached to their pastime [57,58].

Hunters exercise driven hunting in groups of considerable size. In Lower Saxony, Germany, the single hunt was the prevalent method of wild boar hunting [43]. However, drive hunts were increasing and associated with increasing hunting bags. Although single hunts can be efficient, the increasing bag of drive hunts emphasized their high efficiency as well. Single hunts are more efficient in low wild boar densities. On the other hand, drive hunts require a high effort and are not sensible when wild boar densities are low. Instead, they are most efficient in high wild boar densities [43]. These trends suggest that wild boar densities are considerable in the Evros prefecture.

### 4.2. Motivation for Wild Boar Hunting

Wild boar hunters mainly perceive hunting as a recreational activity that increases their contact with nature and wildlife, boosts their physiological and psychological well-being, and bonds them with fellow hunters and their dogs. Hunters from Macedonia and Thrace also highly valued hunting for recreation, emphasizing the physiological, psychological, and social benefits [12]. Similar findings have been reported in studies from North America [15,59,60]. The benefits derived from hunting are similar to those reported by all outdoor recreation participants, both consumptive and non-consumptive [61,62,63]. Contact with nature is the common factor shared by all these activities. The ever-increasing urbanization and demands of everyday life have impeded people from visiting natural landscapes. This has led to the deterioration of human health and well-being, termed ‘nature-deficit disorder’ [64,65]. Hunters seemed to recognize and enjoy these benefits when engaging in outdoor activities, being interested in all the natural elements of the landscape, including game and non-game wildlife [43]. Hunters participate in both consumptive activities other than hunting (e.g., berry picking, fishing) and non-consumptive activities (e.g., birdwatching, hiking) more often than non-hunters [66].

Wild boar hunters enjoy consuming meat. It is common practice among Greek wild boar hunters to skin the kill and hand it over to a local taverna (traditional Greek restaurant) to cook. All group members gather around the table to enjoy the food and share stories about this and other hunting excursions with the locals. Although hunters value meat consumption, they do not consider it an important subsistence for their family. As opposed to other countries [67,68,69], game meat cannot be traded in Greece, therefore it cannot provide income to hunters.

Hunters considered themselves useful as contributors to the local economy and wildlife management. Indeed, they buy guns, ammunition, clothing, dogs and their food, and use transport, accommodation, and local restaurants [14,15]. Also, hunters perceive themselves as stewards of nature [70,71]. They are willing to contribute to wildlife conservation by protecting game species and their habitats [72,73]. In Spain, areas dedicated to hunting have a higher abundance of wild rabbit (*Oryctolagus cuniculus*) and other species, such as the endangered Iberian lynx (*Lynx pardinus*), Spanish imperial eagle (*Aquila adalberti*), and little bustard (*Tetrax tetrax*) [74,75,76]. Further, hunters’ interest in conserving biodiversity indirectly benefits game populations through trophic cascades [77]. Hunting has also been used in wildlife management. For example, hunting has helped to reduce crop damage by regulating overabundant wildlife populations [9,13,78,79]. On the other hand, hunting may also negatively affect wildlife and biodiversity in certain contexts and regions, mainly due to pressure for subsistence, poaching, and poor regulation [80].

Educational level, income, hunter group size, days out hunting, distance covered, and money spent were positively associated with hunting for recreation. In contrast, educational level, income, days out hunting, and distance covered were negatively associated with hunting for its use values. Hunters with a higher income could afford to venture out for more time and greater distances. Having solved their survival and well-being issues, they pursued hunting as a recreational activity. On the other hand, hunters with a lower income could allocate less money to hunting, therefore spending fewer days and traveling closer to home to pursue their favorite activity. The poorer hunters had higher use values, perceiving game meat as important for their family and an important economic activity. Income is generally positively correlated with educational level [81,82], thus explaining the observed relationships of income and educational level with motivations for wild boar hunting. Recreational hunters were strongly committed to wild boar hunting, while use values were unrelated to hunting. Recreation motivations were stronger than utility motivations, which were mediated by income, positively for the former, and negatively for the latter. These factors might explain the observed relationship between motivations and commitment to hunting.

### 4.3. Management Implications

The wild boar hunters of Evros exercised hunting primarily for reaping recreational benefits. However, they were particularly interested in being useful by supporting the local economy and contributing to wildlife management. This interest of hunters in wildlife management and conservation seems to be universal [69,70], producing positive outcomes [9,13,78,79], although not always [80]. Hunters are more highly interested in wildlife management and conservation when game species are involved, which is the case in Evros Prefecture [72,73].

Wild boars may cause considerable damage to crops or pose a significant threat to people and animal health. When this happens, wildlife managers need to apply suitable strategies for reducing their numbers, so that healthy populations are retained, ecosystem function and biodiversity are secured, and hunting quotas are both sufficient and sustainable. However, this is not easy to accomplish. Hunters who hunt for recreation, as in Evros, will want more wild boars, to exercise their favorite activity. This will give rise to conflicts with other key stakeholders, such as cattle farmers, managers, and the general public, who will want wild boar populations to be controlled. Pertinent authorities, such as the Forest Service and the Ministry of the Environment and Energy, must manage the conflict by educating stakeholders, especially hunters, about the need for population control and bringing them together to build trust among the parties. Education and trust are key components of the conflict management process [83]. The acceptance of a management plan by all parties is a prerequisite for its success, with hunters also being important for the implementation of wild boar control strategies. Special care should be given during the conflict management process to those hunters with a low income who hunt for subsistence. Although their numbers are small in Evros, wild boar control may critically restrict their ability to provide for their families. State and local authorities should consider providing financial support to subsistence hunters as a just measure to alleviate their hardship and gain their support for wild boar management [84].

In Greece, strategies such as an unrestricted hunting bag during the winter and the prohibition of the provision of food have been used to control wild boar numbers. However, these strategies have proven ineffective, both in Greece and elsewhere, because of the species’ prolific breeding, the existence of natural refuges, the rugged terrain and dense vegetation, and the reduction in hunters’ numbers [43,85]. A management regime incorporating several strategies adjusted to local conditions has been proposed for effectively regulating wild boar populations [85]. Such strategies can include early piglet hunting in agricultural fields, combined with hunting in forests during the winter [43]. Recreational hunters could be assisted by hired professional hunters who would train and coordinate them, besides carrying out additional hunts. Public administration should also promote regulated hunting, the training of hunters, and facilitating their role so that they can participate in management. Trapping could also be used to complement these strategies and increase efficiency [85]. Corral traps and cages are among the most efficient techniques for reducing wild boar populations [86]. The selection of suitable hunting methods, which is very important for the success of wild boar management, largely depends on the hunters’ knowledge and attitudes, regional geography and vegetation, and wild boar densities [43]. Overall, public opinion, needs, and perceptions of wild boar, along with the needs of the species and the ecosystem, should be considered to allow proper management [87].

## 5. Conclusions

This study investigated the habits and motivation of wild boar hunters in Evros Prefecture, northern Greece. The findings revealed a hunting community strongly attached to their game, dedicating considerable time and effort to pursuing wild boars. The hunters’ main motivation was recreational, while a considerable utility motivation was also present. Sociodemographic characteristics, most notably educational level, income, time, and the expenses devoted to hunting, variously affected motivations. Further research must determine the suitability of hunting methods, investigate the needs and perceptions of all stakeholders, and ultimately propose an integrated management approach for the regulation of the wild boar population. Our findings showed that hunters are willing to participate in wild boar management, the task of balancing many needs, as a critical agent for its effective implementation.

## Figures and Tables

**Table 1:** Motivation, habits, and sociodemographics of wild boar hunters (*n* = 134).

Variable	Definition	Mean	SD	Min	Max
Recreation motivation	Recreation dimension from factor analysis in Table 2 (1 = strongly disagree, 2 = disagree, 3 = neither, 4 = agree, 5 = strongly agree).	4.159	1.144	1	5
Utility motivation	Utility dimension from factor analysis in Table 2 (1 = strongly disagree, 2 = disagree, 3 = neither, 4 = agree, 5 = strongly agree).	3.404	1.111	1	5
Age	Years of age.	42.299	11.306	20	65
Level of education	0 if lower, 1 if higher.	0.254	0.437	0	1
Income	Participant’s annual household income (EUR × 1000).	19.709	16.726	1	100
Hunting experience	How many years have you hunted wild boars? (in years).	15.388	10.178	1	50
Commitment to hunting	If I stop hunting wild boar I will... (1 = not miss it, I have other more important interests, 2 = miss it, but I have other equally important interests, 3 = miss it, but will adjust, 4 = miss it, not replaceable).	3.090	1.179	1	4
Current population trends	Wild boar populations have... (1 = decreased, 2 = remained stable, 3 = increased).	2.381	0.812	1	3
Preferred future population trends	Wild boar populations should... (1 = be reduced, 2 = remain stable, 3 = increase).	2.060	0.839	1	3
Hunters group size	What is the size of your wild boar hunting group? (number of hunters).	8.433	4.678	2	30
Hunting dog ownership	Do you own a hunting dog specializing in wild boar? (0 = no, 1 = yes).	0.843	0.365	0	1
Hunting days	How many days did you go hunting wild boar during the previous hunting season? (number of days).	47.888	15.139	15	69
Nights out	How many nights did you spend outdoors hunting wild boar during the previous hunting season? (number of days).	3.724	8.403	0	40
Travel distance	What was the maximum distance that you traveled for hunting wild boar during the previous hunting season? (in km).	60.261	40.515	10	200
Weekly expenses	What were your weekly expenses for hunting wild boar during the previous hunting season? (in euros).	61.015	36.295	20	200

**Table 2:** Motivation dimensions of wild boar hunters (*n* = 134), as determined by exploratory factor analysis. Descriptive statistics, factor loadings, factor eigenvalues, % variance explained, and factor reliability are given.

**Motive Statements ^a^**	**Mean**	**SD**	**Recreation**	**Utility**
**I Go Wild Boar Hunting Because…**				
It brings me into close contact with nature.	4.321	1.045	*0.709*	−0.023
It offers me excitement.	3.993	1.357	*0.806*	0.005
It helps me stay fit.	3.985	1.354	*0.743*	0.031
It helps me reduce everyday stress.	4.299	1.117	*0.811*	0.081
I enjoy seeing wild boar and other wildlife.	4.306	0.869	*0.609*	0.209
It allows for bonding with my group fellows.	4.216	0.953	*0.626*	−0.101
It allows for bonding with my dogs.	3.993	1.312	*0.812*	0.163
I like the meat.	3.813	0.990	−0.120	*0.707*
The meat is an important subsistence for my family.	1.948	1.356	0.032	*−0.743*
It makes me feel useful to my family.	3.418	0.952	0.091	*0.629*
It helps manage their populations.	3.910	1.093	−0.245	*0.734*
It helps improve the local economy.	3.933	1.165	0.168	*0.583*
Eigenvalue			3.896	2.415
% variance explained			43.311	26.073
Cronbach’s alpha			0.913	0.789

^a^ Range: 1 (strongly disagree)–5 (strongly agree). Factor loadings in italics denote factor membership.

**Table 3:** Relationships of recreation and utility motivations, and preferred future wild boar population trends with habits and sociodemographics of wild boar hunters (*n* = 134).

	**Recreation Motivation**			**Utility Motivation**			**Preferred Future Population Trends**		
	***β***	***z***	***p***	***β***	***z***	***p***	***β***	***z***	***p***
Recreation motivation	−	−	−	−	−	−	0.299	4.052	<0.001 *
Utility motivation	−	−	−	−	−	−	0.285	4.018	<0.001 *
Level of education	0.244	2.736	0.006 *	−0.209	−2.323	0.020 *	−0.275	−3.584	<0.001 *
Income	0.175	2.216	0.027 *	−0.239	−2.546	0.013 *	−0.011	−0.167	0.867
Hunting experience	−0.210	−1.641	0.101	−0.219	−1.694	0.090	−0.385	−3.589	<0.001 *
Commitment to hunting	0.329	3.609	<0.001 *	0.079	0.876	0.381	0.160	2.057	0.040 *
Current population trends	0.078	0.944	0.345	0.272	3.259	0.001 *	−0.524	−7.407	<0.001 *
Hunters group size	0.279	3.26	0.001 *	0.055	0.632	0.527	0.066	0.911	0.362
Hunting dog ownership	−0.073	−0.872	0.383	0.129	1.511	0.131	0.107	1.522	0.128
Hunting days	0.337	4.228	<0.001 *	0.007	0.088	0.930	−0.081	−1.169	0.242
Nights out	0.057	0.619	0.536	−0.301	−3.256	0.001 *	−0.043	−0.549	0.583
Travel distance	0.253	2.613	0.009 *	−0.188	−2.284	0.022 *	−0.126	−1.238	0.198
Weekly expenses	0.347	4.228	<0.001 *	0.141	1.484	0.138	−0.083	−1.019	0.308
Constant	2.499	3.801	<0.001 *	2.896	4.632	<0.001 *	1.936	3.103	0.001 *
adjusted *R*^2^	0.328			0.315			0.547		

* Significant relationships at *p* < 0.05. Note: multiple linear regression models were used. Dummy variables: level of education (lower = 0, higher = 1), hunting dog ownership (no = 0, yes = 1). Standardized regression coefficients, *z*, *p*, and adjusted *R*^2^ values are given.

## Data Availability

The data presented in this study are available on reasonable request from the corresponding author.
